# *Trichoderma* reduces the relative abundance of pathogen-associated fungi while maintaining fungal community stability in *Rhododendron* soils

**DOI:** 10.1093/aobpla/plag051

**Published:** 2026-10-05

**Authors:** Abisola Eniola Folorunso, David J Burke, Sarah R Carrino-Kyker, Jean H Burns

**Affiliations:** Department of Biology, Case Western Reserve University, 10900 Euclid Avenue, Cleveland, OH 44106, United States; The Holden Arboretum, 9500 Sperry Road, Kirtland, OH 44094, United States; The Holden Arboretum, 9500 Sperry Road, Kirtland, OH 44094, United States; Department of Biology, Case Western Reserve University, 10900 Euclid Avenue, Cleveland, OH 44106, United States

**Keywords:** *Rhododendron*, *Trichoderma*, community assembly, soil fungal community, high-throughput sequencing, pathogen suppression

## Abstract

Soil fungal communities are shaped by host-mediated and environmental filters, yet it remains unclear whether microbial inoculants can override these processes. *Trichoderma* is widely used as a biocontrol agent, but its effects on fungal community assembly and functional guild structure in woody plant systems are not well understood. We tested whether *Trichoderma* alters soil fungal communities or acts within host-structured systems to influence functional groups. A factorial greenhouse experiment was conducted using three *Rhododendron* species, four *Trichoderma* treatments (control, *T. asperellum*, *T. harzianum*, and a combined treatment), and four soil treatments (three live conspecific soils and one sterilized soil). Plant performance traits were measured, and soil fungal communities were characterized using internal transcribed spacer 2 high-throughput sequencing. Community composition, alpha diversity, and functional guild abundances were analysed using multivariate and linear modelling approaches. Fungal community composition was structured primarily by host species identity, with soil origin exerting a secondary influence, while *Trichoderma* inoculation had no detectable effect on community composition or diversity. Despite this, *Trichoderma* increased plant relative growth rate and reduced the relative abundance of pathogen-associated fungi in a host-dependent manner, with the strongest reduction observed in *R. yedoense*, while saprotrophic and ectomycorrhizal fungi remained unchanged across treatments. These findings demonstrate that *Trichoderma* does not restructure fungal communities in host-structured soils but instead acts as a functionally selective agent, reducing pathogen-associated fungi under specific ecological contexts. The effectiveness of microbial inoculants therefore depends on host identity and baseline abundance of pathogen-associated fungi, emphasizing the importance of ecological context in shaping plant–microbe interactions.

## Teaser

Beneficial *Trichoderma* fungi are widely used to promote plant health, but their effects on the broader soil fungal community remain less understood. Using three *Rhododendron* species, we found that host species was a major driver of fungal community composition, while *Trichoderma* inoculation did not substantially alter overall fungal community structure or diversity. However, *Trichoderma* reduced the relative abundance of pathogen-associated fungi and enhanced plant growth, suggesting that beneficial fungi can suppress potentially harmful fungi without broadly disrupting resident fungal communities.

## Introduction

Soil fungal communities play a central role in terrestrial ecosystems by mediating nutrient cycling, influencing soil structure, and shaping plant health (Hartmann and Six 2023, Wang *et al.* 2023a). In forested and shrubland systems, the relative abundance of saprotrophic, mutualistic, and pathogenic fungi affects decomposition dynamics, disease pressure, and plant–soil feedbacks. These communities assemble through interacting abiotic, biotic, and host-mediated filters, including soil conditions, dispersal constraints, plant identity, and microbial interactions (Wang *et al.* 2023a). In applied contexts such as horticulture, ecological restoration, and biological control, there is growing interest in whether microbial inoculants can modify these assembly processes in predictable ways (Asghar *et al.* 2024, Guzmán-Guzmán *et al.* 2025).

Current understanding in microbial ecology emphasizes that community assembly is strongly shaped by deterministic filtering, with host identity and environmental conditions acting as dominant ecological filters (Nemergut *et al.* 2013, Vellend 2016). In plant-associated microbiomes, host-driven assembly patterns can remain stable even under short-term environmental change (Fitzpatrick *et al.* 2018, Trivedi *et al.* 2020). This host filtering is especially pronounced in fungal communities, where root traits, exudate chemistry, and litter inputs influence which taxa colonize and persist in the rhizosphere (Berg and Smalla 2009, Philippot *et al.* 2013, Li *et al.* 2022). In woody systems, long-lived roots and accumulated organic matter may further strengthen host-mediated filtering relative to annual systems. Empirical studies show that tree species identity can influence fungal biomass, activity, and richness through effects on soil chemistry and nutrient availability (Heděnec *et al.* 2020, Haq *et al.* 2025). Together, these patterns suggest that host plants may constrain or modulate the effects of introduced microbial inoculants, potentially limiting their ability to override established microbial assemblages.

Among the most widely used fungal inoculants is *Trichoderma*, a genus known for antagonism against plant pathogens, rapid growth, and interactions with plant roots (Yao *et al.* 2023, Guzmán-Guzmán *et al.* 2025). Numerous studies in agricultural systems have demonstrated that *Trichoderma* can suppress soil-borne pathogens, alter nutrient availability, and, in some cases, influence soil microbial communities (Asghar and Kataoka 2021, Sui *et al.* 2022, Asghar *et al.* 2024). For example, *Trichoderma* seed coatings have been shown to reduce *Fusarium* abundance in wheat rhizospheres, accompanied by shifts in fungal community composition (Sui *et al.* 2022). However, most work in this area emphasizes disease suppression or crop yield rather than broader patterns of fungal community assembly.

Despite its widespread application, the extent to which *Trichoderma* reshapes resident microbial communities remains debated. Some studies report substantial shifts in fungal composition or interaction networks following inoculation, often mediated by altered soil chemistry or competitive exclusion (Zhang *et al.* 2019, Wang *et al.* 2023b). In contrast, other work suggests that introduced *Trichoderma* strains persist only transiently, particularly in complex field soils, limiting their capacity to drive lasting community-wide restructuring (Kaminsky *et al.* 2019, Sui *et al.* 2022). These contrasting outcomes highlight a key unresolved question of whether *Trichoderma* acts as a community engineer or primarily as a localized antagonist targeting specific functional groups.

Functional guild composition provides a useful lens for addressing this question. Shifts in the relative abundance of pathogenic, saprotrophic, or mutualistic fungi can alter ecosystem functioning even in the absence of large taxonomic turnover (Tedersoo *et al.* 2014, Nguyen *et al.* 2016). For example, selective reductions in pathogenic fungi could alleviate negative plant–soil interactions and thereby contribute to enhanced plant growth (van Ruijven *et al.* 2020), whereas disruption of dominant saprotrophic guilds could have broader ecosystem consequences (Crowther *et al.* 2012). Distinguishing between guild-level shifts and whole-community restructuring is therefore critical for understanding how microbial inoculants influence plant–soil systems. Plant hosts that strongly structure soil fungal communities provide an ideal context for examining these dynamics.

Rhododendrons are acidophilic woody shrubs with distinct root traits and soil associations, making them a useful system for examining how microbial inoculants interact with host-structured fungal communities (Read 1996, Cairney and Meharg 2003). Despite their ecological and horticultural importance, fungal community dynamics associated with *Rhododendron* species remain poorly characterized, particularly with respect to their root-associated fungi (Hambleton and Currah 1997, Vohník *et al.* 2012). This represents a critical gap in understanding how microbial inoculants function in non-crop, woody plant systems, where host filtering may be especially strong. In this study, we tested how *Trichoderma* inoculation and host species identity shape soil fungal communities and plant performance in *Rhododendron* systems under controlled greenhouse conditions. By combining plant growth measurements with fungal community and functional guild analyses, we asked whether introduced *Trichoderma* strains can override host effects or instead have limited influence in host-structured soils. This approach allowed us to separate direct effects of inoculation from broader host-driven patterns in fungal communities.

Specifically, we aimed to:

1. Quantify the effects of *Trichoderma* species on *Rhododendron* growth dynamics and biomass allocation.
2. Assess the relative importance of host species identity versus *Trichoderma* inoculation in structuring soil fungal community composition.
3. Determine whether *Trichoderma* influences plant–fungal interactions through targeted shifts in functional guilds or whole-community reorganization.

Together, these objectives address whether microbial inoculants act as dominant drivers of soil microbial communities or instead play a secondary role in systems where host identity strongly shapes belowground communities.

## Methods

### Experimental design

A factorial greenhouse experiment was conducted at the Squire Valleevue and Valley Ridge, Case Western Reserve University Farm (Hunting Valley, OH, USA) to test the influence of *Trichoderma* inoculation and soil origin on fungal community structure and *Rhododendron* performance. The design included three *Rhododendron* species (*R. molle*, *R. maximum*, and *R. yedoense*), four *Trichoderma* treatments (control, *T. asperellum*, *T. harzianum*, and a mixed treatment containing both *Trichoderma* species), and four soil treatments (three live field soils representing different *Rhododendron* species and one sterilized soil). Each treatment combination was replicated five times, resulting in 240 pots arranged in 12 randomized blocks within the greenhouse. Two of the five replicates from each treatment combination were randomly selected for soil fungal community sequencing, ensuring that each experimental treatment was represented by two samples, while all five replicates were used for plant performance measurements (see Fig. 1 for experimental workflow and timing of key procedures).

**Figure 1:**
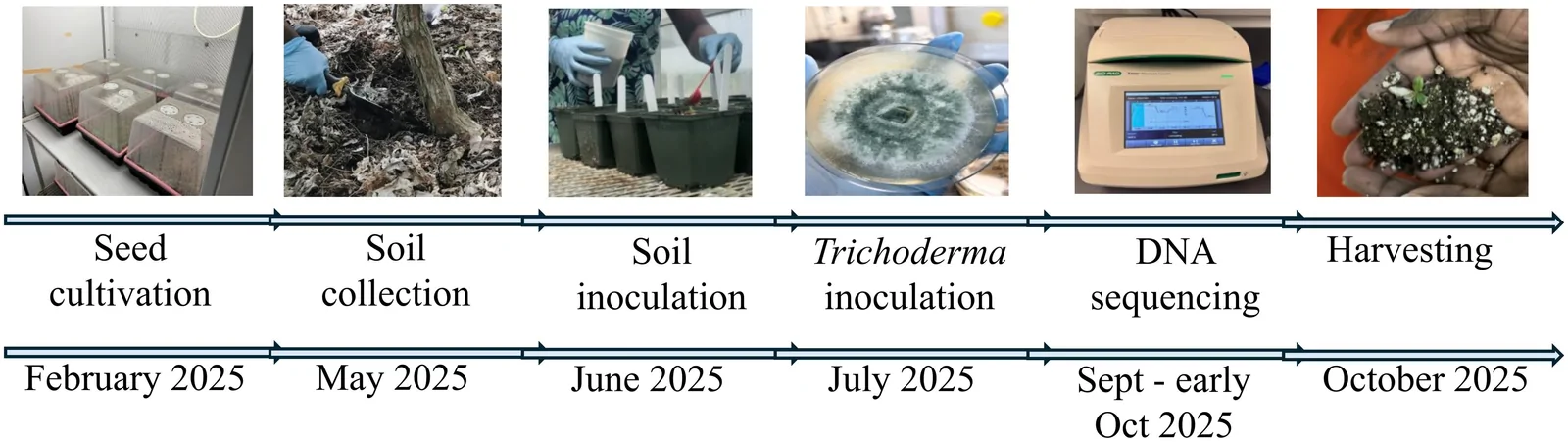
Experimental timeline illustrating the sequence of greenhouse and laboratory procedures.

### Seed collection and cultivation

To prevent unintended hybridization, species-true seeds of three *Rhododendron* species were produced through controlled pollinations. In spring 2022, pollination was carried out at the Holden Arboretum (Kirtland, OH, USA) by enclosing flower buds in mesh bags before anthesis and manually transferring pollen from a different accession of the same species onto receptive stigmas. Mature seed capsules were collected in September 2022 and stored under dry conditions until use. Germination was initiated in February 2025 in controlled-environment growth chambers at the Case Western Reserve University Farm, maintained at a day temperature of 20°C (6 a.m.–10 p.m.) and a night temperature of 15°C. In May 2025, seedlings were transplanted individually into pots (10 cm × 10 cm wide × 9 cm depth) containing a 1:1 mixture of horticultural perlite and peat moss. Each pot contained a single seedling. Seedlings were allowed to establish for 1 month prior to field soil inoculation, to ensure uniform initial growth before exposure to experimental soil communities.

### Field soil collection and preparation

To establish live and sterile soil treatments, rhizosphere soils were collected from beneath mature individuals of the three *Rhododendron* species at the Leach Research Station of the Holden Arboretum (41°49′21″ N, 81°3′10″ W). For each species, soils were sampled from three replicate locations, maintaining independent replicates to avoid pseudo-replication (Reinhart and Rinella 2016). Soils were air-dried in the greenhouse, homogenized, and sieved through a #8 mesh (2.36 mm; Sargent-Welch Scientific Co., IL, USA). Half of each replicate soil was pooled and sterilized by autoclaving at 121°C for 2 h (Tuttnauer 2540EA autoclave, Tuttnauer, Netherlands), while the other half remained unsterilized to serve as live inoculum. Each pot received one teaspoon of live or sterile soil placed as a layer above the base soil in June 2025.

### Trichoderma cultivation and inoculation

Two *Trichoderma* species were selected based on results from our previous study, in which they improved *Rhododendron*’s performance and reduced leaf damage (Folorunso and Burns 2026) as well as their natural abundance in *Rhododendron* rhizosphere soils identified through high-throughput sequencing (Liu *et al.* 2023, Ahmad *et al.* 2025). Cultures of *T. harzianum* Rifai (ATCC 60850) and *T. asperellum* Samuels *et al.* (ATCC 52438) were obtained from the American Type Culture Collection and maintained on yeast mannitol agar (YMA) at 4°C. Cultures were then revived in July 2025 by subculturing onto fresh YMA plates. For inoculation, agar plugs were taken from the actively growing edges of each culture using a sterile 10 mm cork borer and placed about 2 cm below the soil surface near the seedling base. In the combined treatment, one plug of each *Trichoderma* species was positioned on opposite sides of the root zone to allow both species to establish. Inoculations were performed once, 1 month after field soil inoculation.

### Data collection and harvesting

Plant height was recorded as a measure of initial plant size before and after *Trichoderma* inoculation from May 2025 to September 2025. All plants survived until the final harvest. Seedlings were harvested in October 2025. At harvest, each plant was separated into shoot and root components. Roots were carefully rinsed to remove adhering soil and scanned using a WinRhizo root analysis system (EPSON Expression 11000XL scanner) to quantify total root length. Both root and shoot tissues were then oven-dried at 60 °C for a minimum of 10 days and weighed to obtain dry biomass. Plant performance was evaluated using multiple metrics, including relative growth rate (RGR), specific root length (SRL), root-to-shoot ratio (RSR), and total biomass. RGR was calculated from sequential height measurements as (ln *H*_2_ − ln *H*_1_)/(*t*_2_ − *t*_1_), where *H* represents plant height at two time points and *t* represents time (in days). Together, these metrics were used to quantify plant performance across treatments and species and to assess responses to *Trichoderma* inoculation.

### DNA extraction and amplification

Approximately 10 g of rhizosphere soil were aseptically collected from each designated *Rhododendron* replicate pot into sterile plastic bags. Sampling was conducted 8 weeks after *Trichoderma* inoculation to allow sufficient time for microbial establishment and host-mediated effects on fungal community assembly. Samples were then stored at −80°C until DNA extraction. Soil fungal DNA was extracted following the bead-beating protocol as described by Burke (2015). The resulting DNA pellets were resuspended in 100 µl of TE buffer and stored at −20°C in siliconized centrifuge tubes until further use.

Fungal DNA was amplified from the internal transcribed spacer 2 region using primers 58A2F and ITS4 (Burke *et al.* 2005, Martin and Rygiewicz 2005). These primers were selected because they consistently amplify a wide range of fungal taxa and provide reliable species-level resolution (Burke *et al.* 2009). Polymerase chain reactions (PCR) were performed in 25-µl volumes containing 1 µl of template DNA (≈100 ng), 0.2 µM of each primer, 2.0 mM MgCl_2_, 0.8 mM dNTPs, 0.5 µg/µl bovine serum albumin, and 1 unit of FastStart Taq DNA polymerase. Amplification was conducted on a PTC-100 Thermal Cycler (MJ Research, Boston, MA, USA) under the following conditions: initial denaturation at 95°C for 5 min; 30 cycles of 95°C for 30 s, 60°C for 60 s, and 72°C for 60 s; and a final extension at 72°C for 5 min. DNA fragment sizes were verified with agarose gel electrophoresis and band intensities were used to estimate DNA concentration using GelAnalyzer software. The first-round PCR products were then sent to a university Genomics Core Facility. The Genomics Core prepared sequencing libraries from PCR products by adding dual indexes and Illumina adapters, followed by purification and pooled library quality assessment. Sequencing was performed on an Illumina MiSeq platform using a 2 × 300 bp paired-end run, and demultiplexed FASTQ files were returned for downstream analysis.

### High-throughput sequencing

A total of 96 soil samples were sequenced successfully, generating 12 945 948 paired-end reads. Sequence data were processed using the UNOISE pipeline (UNOISE3 algorithm; Edgar 2016a). Forward and reverse reads were merged using the fastq_mergepairs function in USEARCH v11.0.667 (Edgar 2010). Primer sequences were removed using Cutadapt v2.8 (Martin 2011), and low-quality reads were filtered using the fastq_filter command in USEARCH. After merging and quality filtering, 3 986 498 high-quality reads were retained for downstream analyses. These reads were denoised and clustered into zero-radius operational taxonomic units (zOTUs) using the unoise3 algorithm, which removes chimeric sequences and low-abundance variants (<8 reads). Processed reads were then mapped back to the resulting zOTUs using the otu_tab function in USEARCH to generate a sample-by-zOTU abundance table, with 3 216 597 reads assigned to the final table (see Supplementary Table S1). Taxonomic assignments were performed against the UNITE fungal reference database v9.0 (Abarenkov *et al.* 2022) using the SINTAX classifier (Edgar 2016b). To account for variation in sequencing depth among samples, count data were normalized using the estimateSizeFactors function in the DESeq2 package v1.42.1 in R v4.3.1 (R Core Team 2023), following established recommendations for amplicon sequencing data (McMurdie and Holmes 2014).

### Data analysis

Plant performance traits (RGR, shoot biomass, root biomass, total biomass, SRL, RSR) were analysed using factorial linear mixed-effects models implemented with the R package lme4, with *Rhododendron* species, *Trichoderma* treatment, soil origin, and their interactions included as fixed effects, and block included as a random effect. Response variables were log-transformed where necessary to meet model assumptions. Model assumptions (normality and homogeneity of variance) were evaluated using diagnostic plots. Treatment effects and interactions were assessed using type III analysis of variance, and planned contrasts were conducted to evaluate how *Trichoderma* treatments influenced plant performance relative to the control. When significant main effects or interactions were detected, pairwise comparisons were performed using estimated marginal means with Tukey adjustment for multiple comparisons using the R package emmeans.

Fungal community composition was analysed using permutational multivariate analysis of variance (PERMANOVA) based on Bray–Curtis dissimilarity matrices using the R package vegan. Models included species, soil origin, *Trichoderma* treatment, and all two-way interactions among these predictors. Ordination was visualized using non-metric multidimensional scaling (NMDS). Alpha diversity (Shannon diversity) was analysed using linear models with the same fixed effects structure described above. To disentangle direct and indirect effects of *Trichoderma* on plant performance, structural equation models (SEMs) were constructed using the R package lavaan, linking inoculation treatments, fungal community composition (NMDS axes), and plant traits. Models were used to test whether *Trichoderma* effects on plant performance were mediated through shifts in fungal community structure.

Functional guilds were assigned based on taxonomic assignments using FUNGuild v1.1 (Nguyen *et al.* 2016). Only guild assignments with confidence rankings of ‘highly probable’ or ‘probable’ were retained for statistical analyses. The dataset was then subsetted to examine guild assignments that included saprotroph, ectomycorrhizal, and plant pathogen classifications. Although *Rhododendron* species are primarily associated with ericoid mycorrhizal fungi (Luo *et al.* 2025), ectomycorrhizal fungi were sufficiently represented in our sequencing dataset and were therefore retained as a focal guild, whereas ericoid mycorrhizal fungi were not sufficiently represented for separate analysis. The pathogen-associated category refers specifically to fungi assigned to the plant pathogen guild. Note, however, that individual fungal taxa may be assigned to multiple guilds and their ecological functions can vary with environmental conditions and life stage, guild assignments were interpreted as representing potential ecological functions rather than confirmed functional activity (Nguyen *et al.* 2016). Guild-level relative abundances were compared across treatments using linear models with species, soil origin, *Trichoderma* treatment, and their interactions as fixed effects.

## Results

### Effects of *Trichoderma* on plant growth dynamics and biomass allocation

We first examined how *Trichoderma* inoculation influenced plant growth dynamics and biomass allocation across *Rhododendron* species (see Supplementary Table S2). RGR varied among *Rhododendron* species (*F*_2,192_ = 48.87, *P* < .001) and increased under *Trichoderma* inoculation (*F*_3,192_ = 31.98, *P* < .001; Fig. 2). The effect of *Trichoderma* also differed among *Rhododendron* species, as indicated by a significant Species × *Trichoderma* interaction (*F*_6,192_ = 4.51, *P* < .001). Pairwise comparisons indicated higher RGR under *T. asperellum* than under the control in *R. maximum* (*t*_192_ = 4.86, *P* < .0001) and *R. yedoense* (*t*_192_ = 4.90, *P* < .0001). Similarly, RGR was higher under *T. harzianum* than under the control in *R. maximum* (*t*_192_ = 6.43, *P* < .0001) and *R. yedoense* (*t*_192_ = 5.05, *P* < .0001), whereas neither treatment differed significantly from the control in *R. molle*. RGR did not differ between *T. asperellum* and *T. harzianum* within any host species. Soil origin did not affect RGR, and interactions involving soil were not significant (Supplementary Table S2), indicating that these effects were consistent across soil environments. Biomass patterns were dominated by host identity, with shoot biomass (*F*_2,190.44_ = 75.45, *P* < .001), root biomass (*F*_2,192_ = 117.99, *P* < .001), and total biomass (*F*_2,190.00_ = 91.30, *P* < .001) differing significantly among *Rhododendron* species. In contrast, *Trichoderma* and soil treatments had no significant effects on these biomass traits, and no significant interactions were detected (Supplementary Table S2).

**Figure 2:**
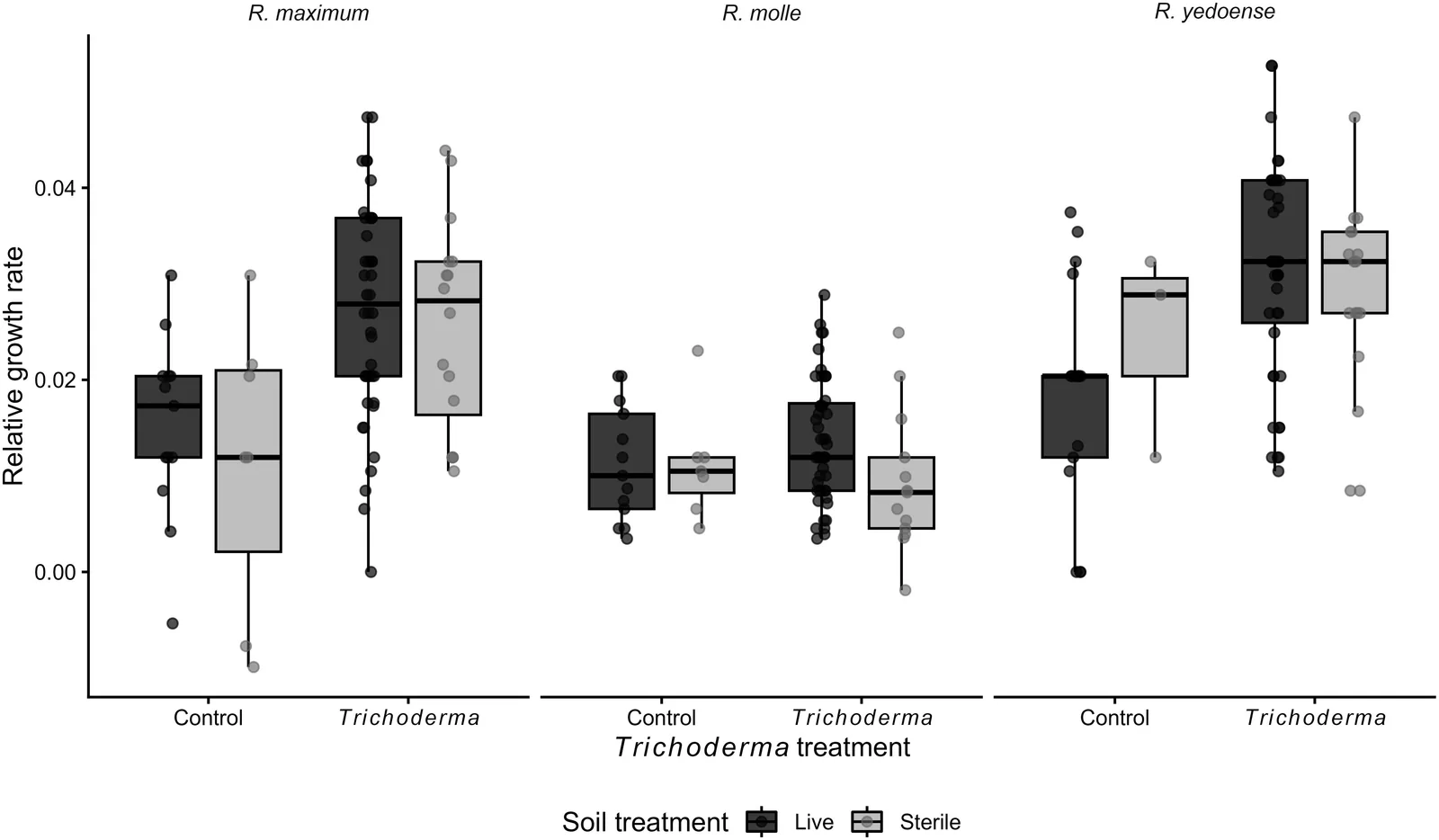
Relative growth rate (RGR) of *Rhododendron* species under *Trichoderma* inoculation and soil treatments (live vs. sterile). Boxplots represent the median and interquartile range, with individual observations shown as points.

Allocation traits were also primarily species-dependent. SRL differed among host species (*F*_2,188.62_ = 21.61, *P* < .001) and showed a significant species × soil interaction (*F*_6,190.09_ = 2.26, *P* = .039). Pairwise comparisons indicated that, for *R. yedoense*, SRL was higher in live2 (*t*_190_ = 3.01, *P* = .016) and live3 soils (*t*_192_ = 3.67, *P* = .002) than in sterilized soil whereas no differences among soil treatments were detected for *R. maximum* or *R. molle*. *Trichoderma* had no significant effect on SRL. RSR also varied among species (*F*_2,192_ = 66.51, *P* < .001), while *Trichoderma* showed a marginal effect on RSR (*F*_3,192_ = 2.38, *P* = .071). Soil treatment and all interaction effects were not significant (Supplementary Table S2).

### Drivers of fungal community composition

We next assessed the relative contributions of host identity, soil origin, and *Trichoderma* inoculation to fungal community composition. Fungal community composition was structured primarily by host species identity, as shown by NMDS ordination (Fig. 3). PERMANOVA analyses revealed a strong species effect (*R*^2^ = 0.191, *F* = 12.17, *P* < .001). Soil treatment explained a smaller but significant proportion of variance (*R*^2^ = 0.041, *F* = 1.72, *P* = .030), and a species × soil interaction was detected (*R*^2^ = 0.088, *F* = 1.86, *P* = .003), indicating host-dependent soil legacy effects. In contrast, *Trichoderma* inoculation did not significantly alter overall community composition (*R*^2^ = 0.033, *F* = 1.41, *P* = .105), and no interactions involving *Trichoderma* inoculation were detected. Thus, variation in fungal assemblages was largely attributable to host filtering and, to a lesser extent, soil origin. Genus-level composition of fungal communities, including the ten most abundant taxa across treatments, is shown in Fig. 4. The most abundant genera across treatments were largely saprotrophic, indicating that rhizosphere fungal communities were broadly dominated by decomposer-associated taxa. Within this overall pattern, host species strongly structured genus-level composition: *R. maximum* and *R. molle* soils were characterized by high dominance of *Gymnopilus* and *Trichomonascus*, whereas *R. yedoense* soils exhibited a more even distribution of genera, including *Peniophorella*, *Ascocorticium*, *Mortierella*, and *Umbelopsis*. Patterns of alpha diversity were consistent with multivariate results. Shannon diversity differed among *Rhododendron* species (*F*_2,72_ = 8.58, *P* = .00046) and between soil treatments (live vs. sterile; *F*_1,72_ = 11.20, *P* = .0013), with a significant species × soil interaction (*F*_2,72_ = 3.50, *P* = .035). In contrast, *Trichoderma* treatment had no effect on alpha diversity (*F*_3,72_ = 1.48, *P* = .227) and did not interact with species or soil (all *P* > .30).

**Figure 3:**
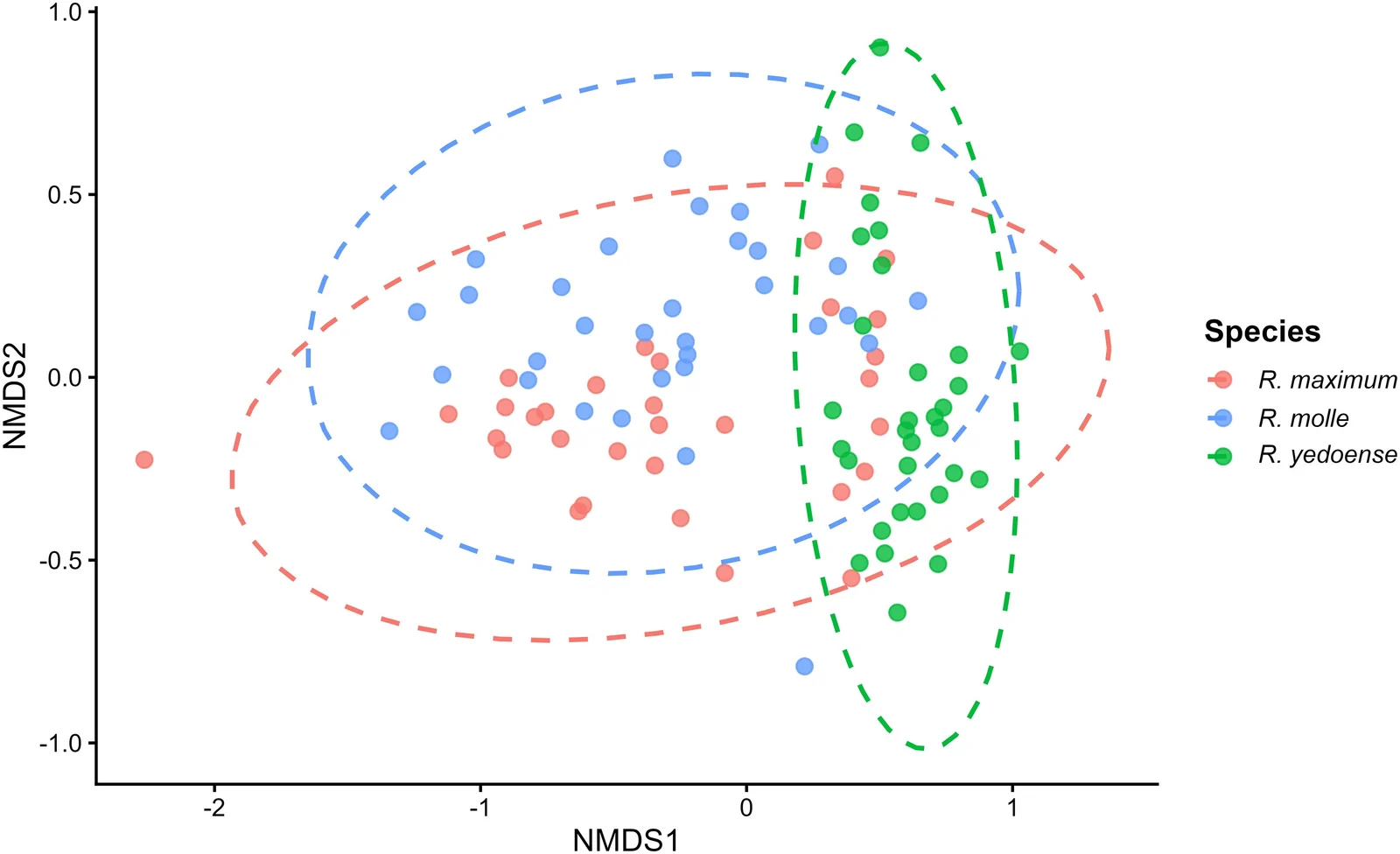
NMDS ordination of soil fungal community composition across *Rhododendron* species. Points represent individual samples and are coloured by host species (*R. maximum*, *R. molle*, *R. yedoense*). Ellipses indicate 95% confidence intervals around species centroids.

**Figure 4:**
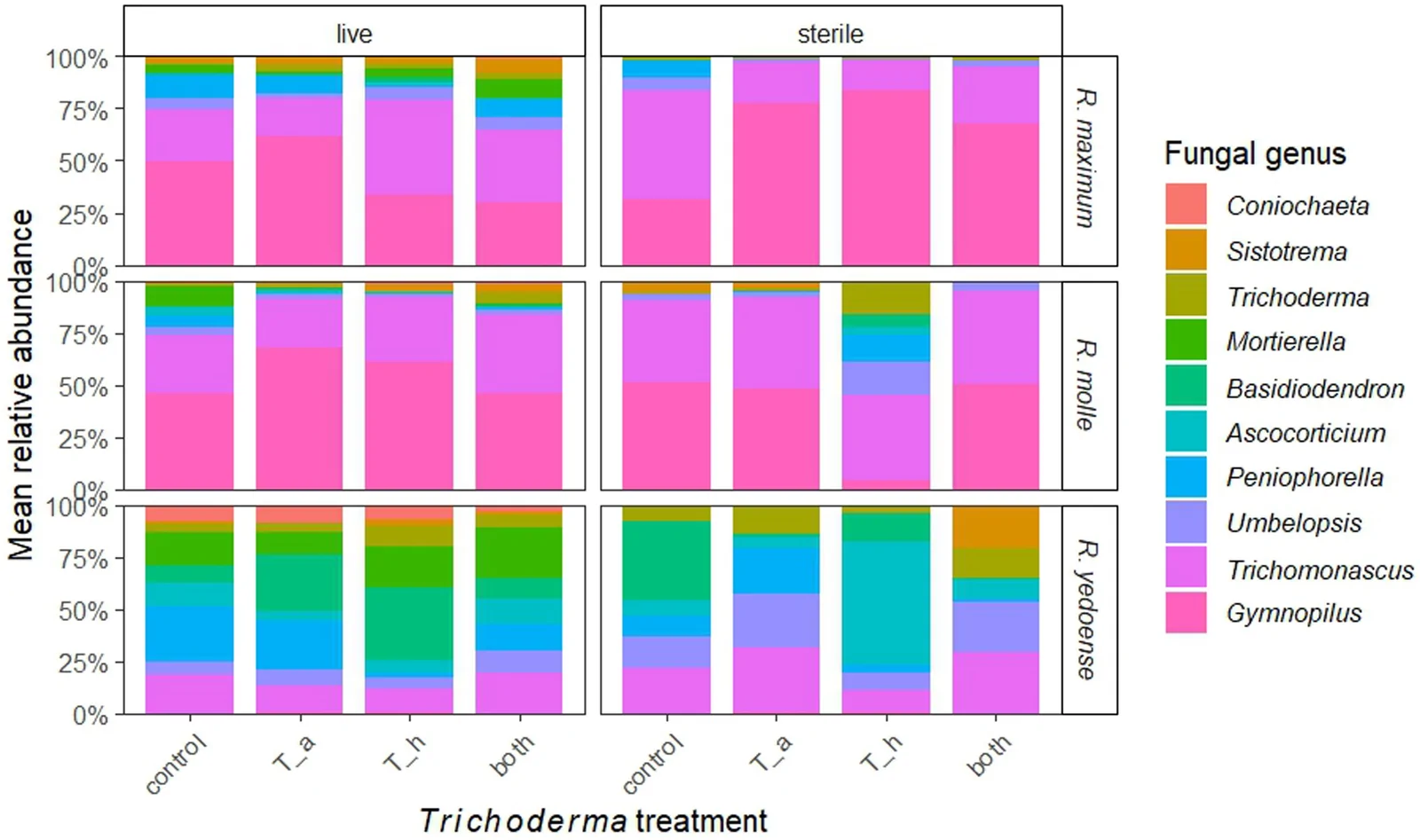
Relative abundance of the ten most abundant fungal genera across *Rhododendron* species, soil treatments, and *Trichoderma* inoculation treatments. Bars represent mean relative abundance within each treatment group. T_a = *T. asperellum*; T_h = *T. harzianum*; both = combined inoculation with *T. asperellum* and *T. harzianum*.

### Direct versus community-mediated effects of *Trichoderma*

To determine whether plant responses were driven by shifts in fungal community structure, we evaluated pathways linking inoculation, community composition, and plant traits (Fig. 5a). Despite measurable effects on plant growth, *Trichoderma* treatments were not associated with broad shifts in fungal community structure. SEMs detected no indirect effects of *Trichoderma* on plant traits mediated through the dominant community axis (NMDS1; all *P* > .09). Likewise, community ordination scores did not significantly predict biomass or allocation traits. However, SEMs revealed several direct effects of inoculation on plant performance. Both *Trichoderma* species were positively associated with RGR (both *P* < .001), while most biomass and allocation traits showed minimal or inconsistent responses across species. Soil treatments exhibited similarly limited direct effects, with only sterile soil showing a negative association with RGR (*P* = .037). Host identity exerted the strongest effects across models. Relative to *R. maximum, R. molle* showed lower RGR but higher total, shoot, and root biomass, as well as higher RSRs and lower SRL (all *P* < .001). In contrast, *R. yedoense* exhibited a weaker pattern, with only a positive association with NMDS1 and a higher RSR (*P* = .023). Collectively, no indirect pathways linking inoculation to plant performance through fungal community composition were supported, indicating that plant responses to *Trichoderma* were driven primarily by direct effects and strong host-dependent differences rather than community-mediated mechanisms (Fig. 5b).

**Figure 5:**
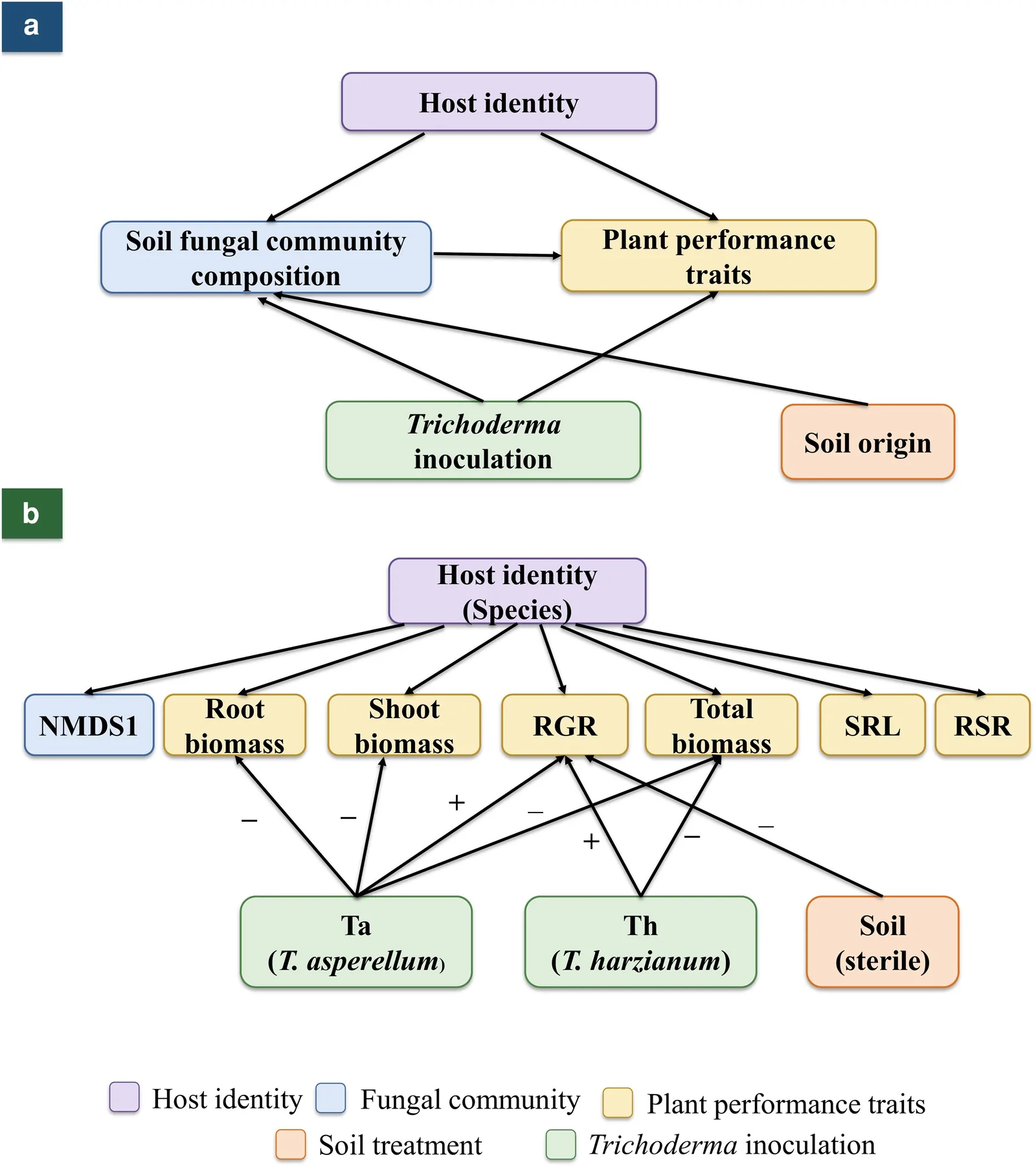
Structural equation models showing (a) hypothesized relationships among host identity, soil treatment, *Trichoderma* inoculation, fungal community composition, and plant traits, and (b) significant pathways (*P* < .05) in the simplified model. Positive (+) and negative (−) signs indicate the direction of effects. Ta, *T. asperellum*; Th, *T. harzianum*. Species effects vary among host plants and therefore do not have a consistent direction.

### Functional guild responses to *Trichoderma* inoculation

Finally, we tested whether *Trichoderma* altered plant–fungal interactions through shifts in functional guild composition rather than whole-community restructuring. Guild-level analyses revealed that *Trichoderma* effects were restricted to specific functional groups rather than the fungal community as a whole. The most pronounced response occurred within pathogenic fungi, where relative abundance declined under *Trichoderma* inoculation, but this pattern was strongly host-dependent. In particular, *R. yedoense* exhibited high pathogen loads under control conditions that were reduced by ∼30% following *Trichoderma* inoculation, whereas the relative abundance of plant pathogen-associated fungi in *R. maximum* and *R. molle* remained comparatively low and showed no detectable response to *Trichoderma* inoculation (Fig. 6). In contrast, saprotrophic fungi did not vary across *Trichoderma*, soil, or host species treatments (all *P* > .18). Relative abundance consistently exceeded 0.9 across all host species, soils, and inoculation treatments, with no detectable shifts under *Trichoderma* application (Fig. 7). This stability indicates that the dominant decomposer component of the fungal community was not disrupted by inoculation. Ectomycorrhizal fungi showed no significant main effects of *Trichoderma*, soil, or host species (all *P* ≥ .21), but a significant soil × species interaction was detected (*P* = .045), indicating that responses to soil treatments varied among host species. Together, these results indicate that *Trichoderma* effects were functionally selective, reducing pathogen abundance in a host-dependent manner while leaving dominant saprotrophic and ectomycorrhizal groups largely unchanged.

**Figure 6:**
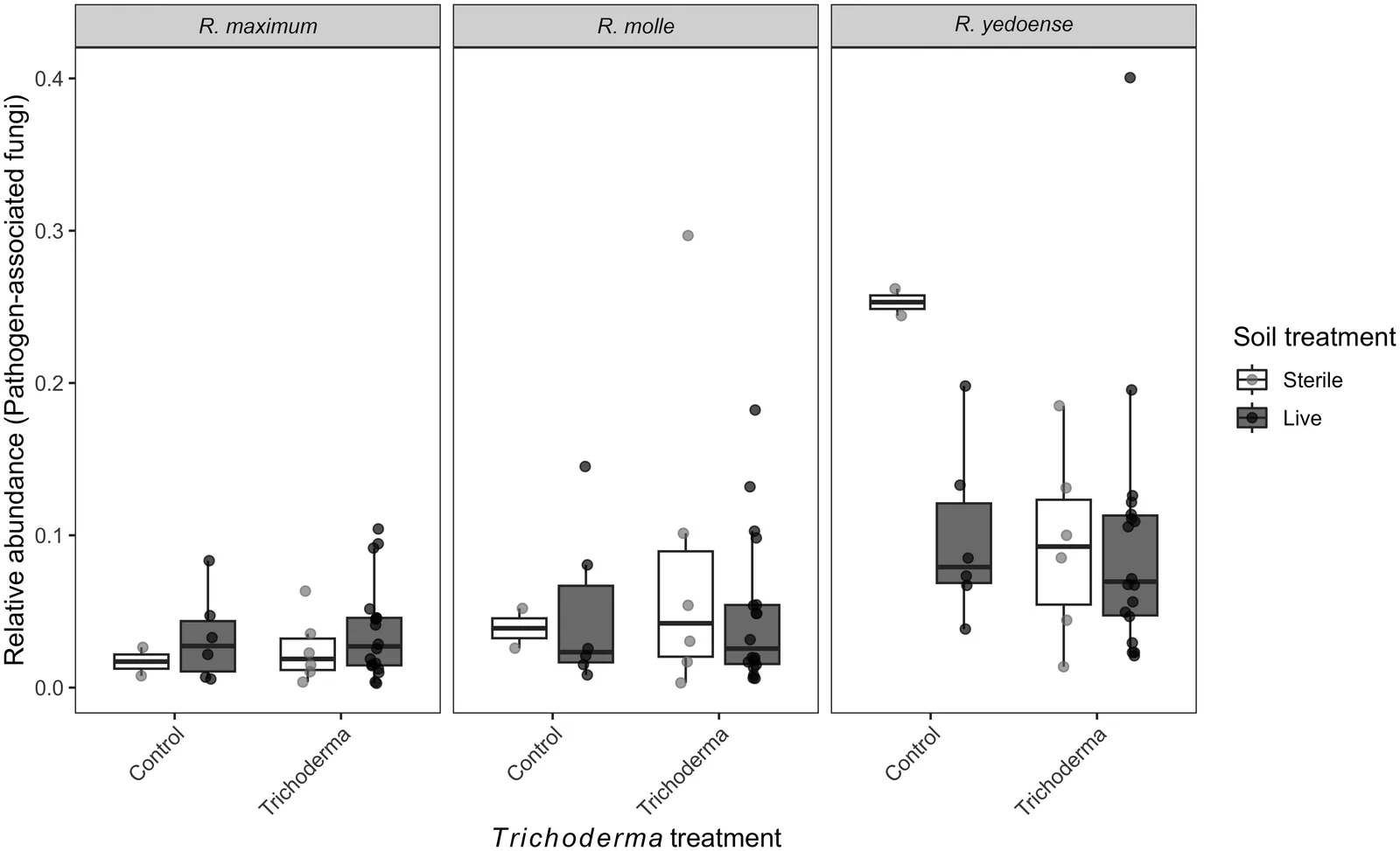
Relative abundance of pathogen-associated fungi across *Trichoderma* treatments, soil treatments, and *Rhododendron* species. Boxplots show the median and interquartile range, and points represent individual observations.

**Figure 7:**
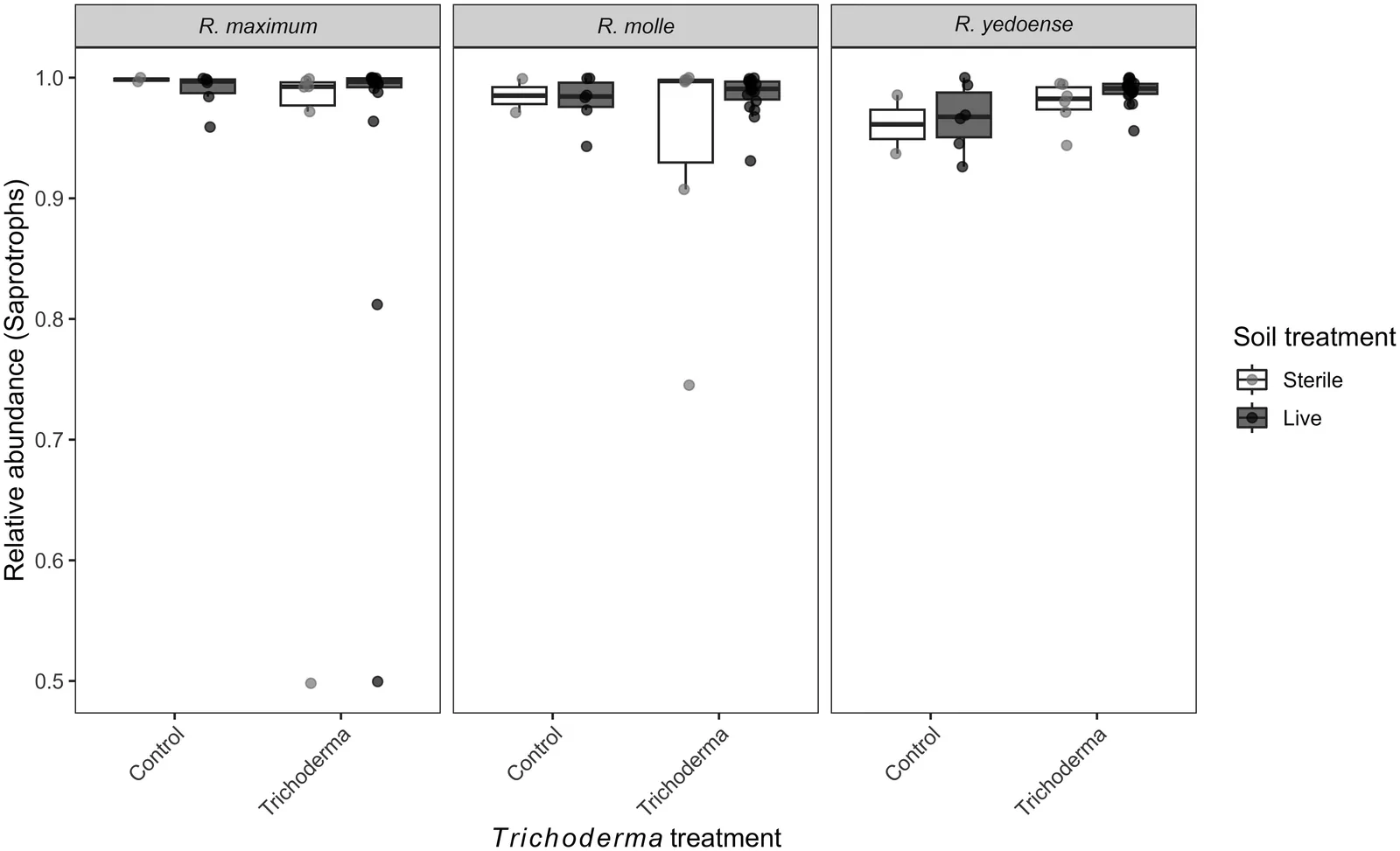
Relative abundance of saprotrophic fungi across *Trichoderma* treatments, soil treatments, and *Rhododendron* species. Boxplots show the median and interquartile range, and points represent individual observations.

## Discussion

Fungal community assembly in *Rhododendron* soils was governed primarily by host species identity, with *Trichoderma* acting as a context-dependent modifier rather than a dominant driver of community structure. Consistent with these community-level patterns, *Trichoderma* altered plant growth dynamics by increasing RGR without corresponding increases in biomass. This decoupling suggests that *Trichoderma* primarily influences early growth processes rather than cumulative biomass accumulation. Differences between growth rate and biomass production can arise from shifts in resource allocation and transient physiological responses (Lambers *et al.* 2008, Poorter *et al.* 2012). One possible explanation is that *Trichoderma* enhances nutrient uptake efficiency and root activity, leading to rapid initial growth without sustained allocation to structural tissues. In addition, *Trichoderma* produces auxin-like compounds and other growth regulators that stimulate root development and plant growth (Gravel *et al.* 2007, Contreras-Cornejo *et al.* 2009, Yao *et al.* 2023), potentially accelerating short-term growth responses that do not necessarily translate into higher final biomass.

In addition to its effects on plant growth, *Trichoderma* reduced the relative abundance of plant pathogen-associated fungi, but this effect was strongly dependent on host species identity, with the clearest response observed in *R. yedoense*. This pattern suggests that the effects of *Trichoderma* on plant pathogen-associated fungi may depend on host-specific microbial contexts. Mechanistically, pathogen suppression by *Trichoderma* is well supported and includes direct antagonism through mycoparasitism and the production of antifungal secondary metabolites such as peptaibols and gliotoxin, as well as indirect effects via induction of plant defence responses (Harman *et al.* 2004, Vinale *et al.* 2008, Shoresh *et al.* 2010). The pronounced reduction in the relative abundance of plant pathogen-associated fungi in *R. yedoense* may reflect higher baseline pathogen pressure or greater host susceptibility, creating conditions under which *Trichoderma*-mediated suppression is more effective (Folorunso and Burns 2026). However, because pathogen activity and disease severity were not measured in this study, these mechanisms remain hypotheses requiring further investigation. Variation in host susceptibility and pathogen performance is widely recognized as a key driver of disease dynamics and microbial community structure (Tack *et al.* 2012, Nemergut *et al.* 2013). In contrast, the relatively low abundance of pathogen-associated fungi in *R. maximum* and *R. molle* may provide less opportunity for detectable *Trichoderma*-associated reductions, consistent with evidence that disease outcomes and pathogen abundance depend strongly on underlying environmental and host-related conditions (Vellend 2016, Vicente-Santos *et al.* 2023, Lahlali *et al.* 2024).

At the community level, fungal composition was structured primarily by host species identity, with soil origin playing a secondary role and *Trichoderma* inoculation having no detectable effect. These results support the view that plant hosts act as strong ecological filters, shaping microbial communities through root traits, exudate chemistry, and litter inputs (Berg and Smalla 2009, Philippot *et al.* 2013, Trivedi *et al.* 2020). In woody systems such as *Rhododendron*, long-lived root systems and persistent organic matter inputs may further stabilize host-associated microbial assemblages (Wardle *et al.* 2004, Urbanová *et al.* 2015). Consistent with previous studies, host identity explained a substantial proportion of variation in fungal community composition, often exceeding the influence of environmental variation or short-term perturbations (Peay *et al.* 2016, Fitzpatrick *et al.* 2018). Together, these findings highlight host filtering as a dominant driver of fungal community assembly in *Rhododendron* soils (Ahmad *et al.* 2025). Because this experiment was conducted using young seedlings, host-driven effects on fungal community assembly were detected at an early developmental stage. As root systems expand and rhizosphere interactions intensify with plant age, these host-mediated filtering effects are likely to become even more pronounced in mature, established individuals.

The absence of a detectable effect of *Trichoderma* on overall community composition suggests that introduced strains did not override existing microbial structure. This aligns with evidence that microbial inoculants often exhibit limited community-wide influence in complex soils due to strong biotic interactions and niche pre-emption by resident taxa (Kaminsky *et al.* 2019, Sui *et al.* 2022). Despite differences in community diversity between soil types, *Trichoderma* sequences were recovered from both sterile and live soils (Fig. 8), indicating that resident soil microbial communities did not prevent establishment of the inoculated strains. Together, these results show that *Trichoderma* influences plant performance primarily through direct interactions rather than through broad restructuring of fungal community composition.

**Figure 8:**
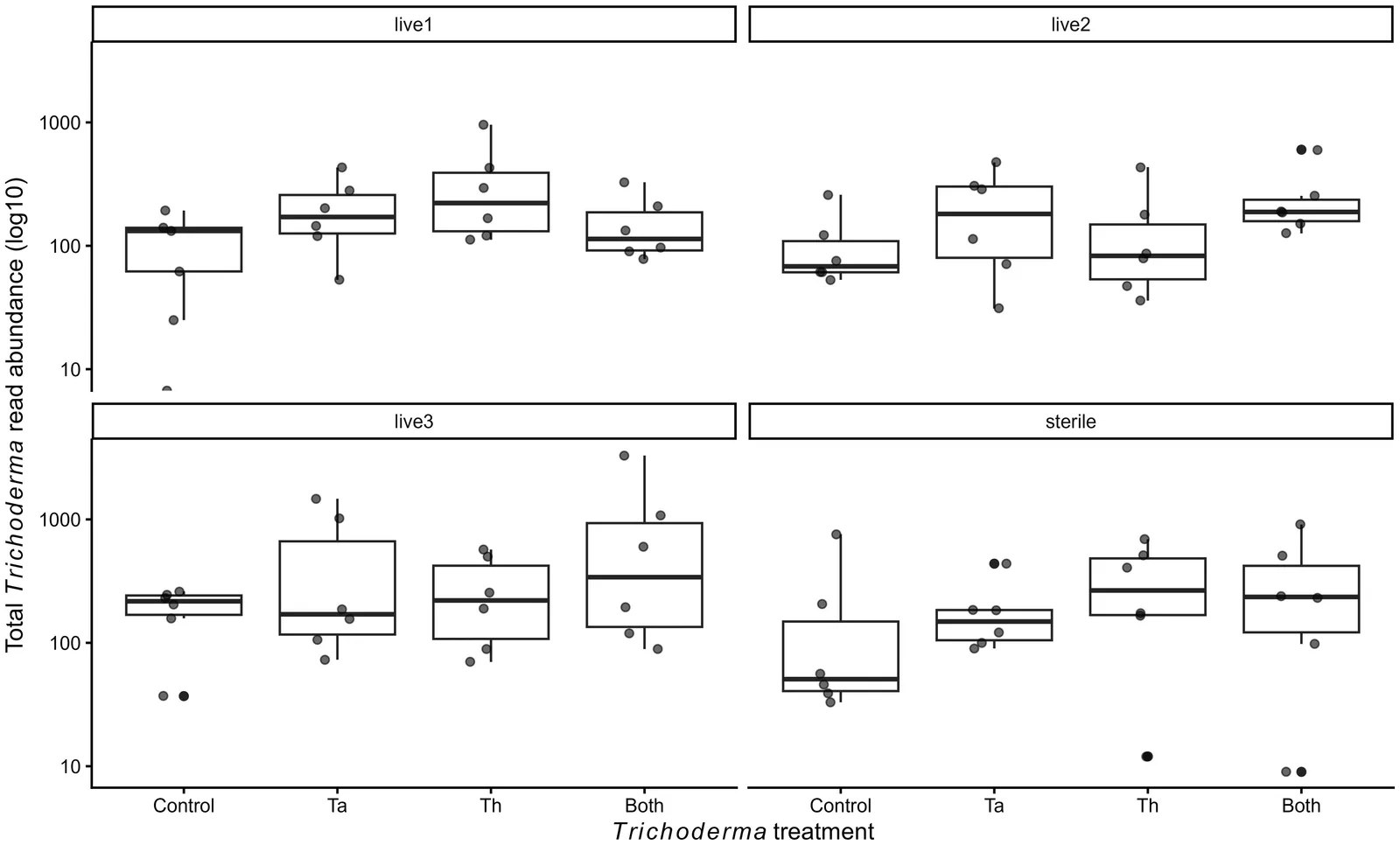
Relative abundance of *Trichoderma* sequences across experimental treatments. *Trichoderma* treatment had a significant overall effect on read abundance (*P* = .026), whereas pairwise comparisons within individual soil treatments were not significant. Ta = *T. asperellum*; Th = *T. harzianum*; Both = combined inoculation with *T. asperellum* and *T. harzianum*.

Rather than producing broad community-wide changes, *Trichoderma* was associated with selective changes in functional guild composition, particularly a reduction in the relative abundance of plant pathogen-associated fungi in one *Rhododendron* species while leaving the dominant saprotrophic group largely unchanged. This functional specificity suggests that key ecosystem processes such as decomposition and nutrient cycling remain intact following inoculation. This has important implications for the application of biocontrol agents in woody and perennial systems, where stable plant–soil feedbacks may limit large-scale community restructuring (Wardle *et al.* 2004, Bever *et al.* 2010, 2012). Consequently, effective use of *Trichoderma* may depend on matching inoculant function to ecological context, particularly across hosts and microbial communities that differ in the baseline abundance of pathogen-associated fungi, rather than expecting broad shifts in microbial community composition (Harman *et al.* 2004, Kaminsky *et al.* 2019).

Collectively, these results show that the effects of *Trichoderma* in *Rhododendron* soils are shaped by the existing ecological context, particularly host species identity and background pathogen dynamics. While *Trichoderma* enhanced plant growth rates and reduced the relative abundance of pathogen-associated fungi, these effects were selective and did not extend to broad changes in fungal community composition. This indicates that microbial inoculation operates within, rather than overriding, established host–microbe interactions. The distinction between functional shifts and compositional stability highlights the importance of evaluating microbial inoculants beyond community-level metrics alone. Future work should therefore determine whether reductions in the relative abundance of plant pathogen-associated fungi translate into reduced pathogen activity or disease and identify the ecological conditions under which these responses occur.

## Supplementary Material

plag051_Supplementary_Data

## Data Availability

Data are archived for peer review at the Open Science Framework: https://doi.org/10.17605/OSF.IO/U6TSA. The sequencing data are also archived in the Sequence Read Archive under BioProject number PRJNA1458291| Fungal microbiome of *Rhododendron* soil with *Trichoderma* inoculation, at the National Center for Biotechnology Information.
