# Supplementary material for: Trichoderma reduces the relative abundance of pathogen-associated fungi while maintaining fungal community stability in Rhododendron soils
Source: AoB Plants. 2026 Oct 5;18(5):plag051. doi: 10.1093/aobpla/plag051 (PMC13671402; doi:10.1093/aobpla/plag051)
Supplement: Supplementary file 1 [file plag051_supplementary_data.pdf]

Title: *Trichoderma* reduces the relative abundance of pathogen-associated fungi while maintaining fungal community stability in *Rhododendron* soils

Table S1: Sequencing read counts for all samples used in fungal community analyses

Sample_ID	Species	soil_trt	Trich_trt	Final_reads
max1	*R. maximum*	sterile	T_h	45631
max10	*R. maximum*	live1	T_a	50158
max15	*R. maximum*	sterile	T_a	41791
max17	*R. maximum*	live3	T_a	32216
max19	*R. maximum*	live3	T_h	24686
max24	*R. maximum*	sterile	control	48050
max26	*R. maximum*	sterile	T_a	43608
max27	*R. maximum*	live1	T_a	31035
max3	*R. maximum*	sterile	both	45568
max31	*R. maximum*	live2	T_h	40144
max32	*R. maximum*	live2	both	37283
max34	*R. maximum*	live3	T_a	35432
max35	*R. maximum*	live2	control	36849
max37	*R. maximum*	live3	both	45498
max38	*R. maximum*	live3	T_h	38930
max40	*R. maximum*	sterile	T_h	45603
max43	*R. maximum*	sterile	control	32016
max47	*R. maximum*	live1	both	33740
max48	*R. maximum*	live1	T_h	27695
max5	*R. maximum*	sterile	both	49855
max51	*R. maximum*	live2	control	52643
max52	*R. maximum*	live2	T_h	37149
max53	*R. maximum*	live2	both	40954
max57	*R. maximum*	live3	control	11814
max58	*R. maximum*	live3	control	34707
max66	*R. maximum*	live1	T_h	37939
max68	*R. maximum*	live1	control	38739
max7	*R. maximum*	live1	both	49069

max72	*R. maximum*	live2	T_a	22658
max73	*R. maximum*	live2	T_a	15205
max77	*R. maximum*	live3	both	29105
max8	*R. maximum*	live1	control	43647
mol102	*R. molle*	sterile	control	34039
mol103	*R. molle*	sterile	T_h	24337
mol104	*R. molle*	sterile	T_a	53066
mol107	*R. molle*	live1	T_a	66104
mol108	*R. molle*	live1	both	36937
mol111	*R. molle*	live2	T_a	34749
mol121	*R. molle*	sterile	both	63292
mol127	*R. molle*	live1	both	52114
mol130	*R. molle*	live1	T_h	47522
mol132	*R. molle*	live2	control	47457
mol133	*R. molle*	live2	T_h	40702
mol138	*R. molle*	live3	T_h	55840
mol139	*R. molle*	live3	control	16449
mol140	*R. molle*	live3	both	29096
mol143	*R. molle*	sterile	both	37363
mol144	*R. molle*	sterile	T_h	26779
mol147	*R. molle*	live1	control	52028
mol148	*R. molle*	live1	T_h	28706
mol150	*R. molle*	live1	control	47333
mol152	*R. molle*	live2	control	50618
mol153	*R. molle*	live2	T_a	32863
mol155	*R. molle*	live2	both	37379
mol156	*R. molle*	live3	control	32508
mol157	*R. molle*	live3	T_a	39402
mol82	*R. molle*	sterile	T_a	25273
mol83	*R. molle*	sterile	control	49354
mol87	*R. molle*	live1	T_a	51930
mol93	*R. molle*	live2	T_h	39436
mol94	*R. molle*	live2	both	40489

mol96	*R. molle*	live3	both	54562
mol98	*R. molle*	live3	T_a	60455
mol99	*R. molle*	live3	T_h	25996
yed162	*R. yedoense*	sterile	T_a	17431
yed163	*R. yedoense*	sterile	both	48325
yed167	*R. yedoense*	live1	T_a	27480
yed168	*R. yedoense*	live1	T_h	29426
yed169	*R. yedoense*	live1	both	18341
yed171	*R. yedoense*	live2	control	23193
yed176	*R. yedoense*	live3	control	27680
yed177	*R. yedoense*	live3	both	21792
yed182	*R. yedoense*	sterile	both	1360
yed183	*R. yedoense*	sterile	T_h	28780
yed187	*R. yedoense*	live1	control	27379
yed188	*R. yedoense*	live1	T_a	21118
yed191	*R. yedoense*	live2	T_h	24020
yed193	*R. yedoense*	live2	both	26347
yed197	*R. yedoense*	live3	T_a	24399
yed202	*R. yedoense*	sterile	control	24305
yed203	*R. yedoense*	sterile	T_h	21329
yed204	*R. yedoense*	sterile	control	10207
yed207	*R. yedoense*	live1	T_h	16079
yed208	*R. yedoense*	live1	control	216
yed211	*R. yedoense*	live2	both	23402
yed213	*R. yedoense*	live2	T_h	36093
yed215	*R. yedoense*	live3	both	30421
yed218	*R. yedoense*	live3	T_h	21262
yed219	*R. yedoense*	live3	T_h	17014
yed222	*R. yedoense*	sterile	T_a	15589
yed226	*R. yedoense*	live1	both	6529
yed231	*R. yedoense*	live2	control	22351
yed232	*R. yedoense*	live2	T_a	4948
yed234	*R. yedoense*	live2	T_a	17770

yed237	*R. yedoense*	live3	T_a	22925
yed238	*R. yedoense*	live3	control	25491

Notes:

T_a = *Trichoderma asperellum*

T_h = *Trichoderma harzianum*

both = *Trichoderma asperellum* + *Trichoderma harzianum*

Table S2. Results of linear mixed-effects models testing the effects of *Rhododendron* species, *Trichoderma* treatment, soil treatment, and their interactions on plant performance traits. Block was included as a random effect.

Plant trait	Effect	Num df	Den df	F-value	P-value
**Shoot biomass**	Species	2	190.444	**75.451**	**<0.001**
	Trichoderma	3	8.509	1.072	0.411
	Soil	3	189.629	0.822	0.483
	Species × *Trichoderma*	6	190.159	0.675	0.670
	Species × Soil	6	188.260	0.970	0.447
	Trichoderma × Soil	9	188.511	0.961	0.474
	Species × *Trichoderma* × Soil	18	187.760	1.477	0.102
**RGR**	Species	2	192	**48.868**	**<0.001**
	Trichoderma	3	192	**31.982**	**<0.001**
	Soil	3	192	0.080	0.971
	Species × *Trichoderma*	6	192	**4.508**	**<0.001**
	Species × Soil	6	192	0.628	0.708
	Trichoderma × Soil	9	192	0.752	0.660
	Species × *Trichoderma* × Soil	18	192	0.868	0.618
**Root biomass**	Species	2	192	**117.990**	**<0.001**
	Trichoderma	3	192	1.891	0.133
	Soil	3	192	0.629	0.597
	Species × *Trichoderma*	6	192	0.768	0.596
	Species × Soil	6	192	1.108	0.359
	Trichoderma × Soil	9	192	0.510	0.866
	Species × *Trichoderma* × Soil	18	192	1.144	0.313
**SRL**	Species	2	188.619	**21.610**	**<0.001**
	Trichoderma	3	8.495	1.423	0.302

	Soil	3	189.578	1.160	0.326
	Species × *Trichoderma*	6	188.558	1.266	0.275
	Species × Soil	6	190.089	**2.263**	**0.039**
	Trichoderma × Soil	9	188.950	1.349	0.214
	Species × *Trichoderma* × Soil	18	189.553	1.145	0.312
**Total biomass**	Species	2	189.996	**91.299**	**<0.001**
	Trichoderma	3	8.284	1.027	0.429
	Soil	3	189.649	0.663	0.576
	Species × *Trichoderma*	6	189.783	0.647	0.692
	Species × Soil	6	188.977	0.929	0.476
	Trichoderma × Soil	9	188.693	0.836	0.584
	Species × *Trichoderma* × Soil	18	188.451	1.404	0.134
**RSR**	Species	2	192	**66.505**	**<0.001**
	Trichoderma	3	192	2.378	0.071
	Soil	3	192	1.620	0.186
	Species × *Trichoderma*	6	192	1.883	0.086
	Species × Soil	6	192	0.862	0.524
	Trichoderma × Soil	9	192	0.875	0.548
	Species × *Trichoderma* × Soil	18	192	1.124	0.332
